# Evaluation of the stability of ceftazidime/avibactam in elastomeric infusion devices used for outpatient parenteral antimicrobial therapy utilizing a national stability protocol framework

**DOI:** 10.1093/jacamr/dlae056

**Published:** 2024-04-05

**Authors:** Saiyuri Naicker, Jason A Roberts, Hayoung Won, Steven C Wallis, Sean Unwin, Conor Jamieson, Tim Hills, Mark Gilchrist, Mark Santillo, R Andrew Seaton, Felicity Drummond, Fekade B Sime

**Affiliations:** The University of Queensland Centre for Clinical Research, University of Queensland, Brisbane, Australia; The University of Queensland Centre for Clinical Research, University of Queensland, Brisbane, Australia; Herston Infectious Diseases Institute (HeIDI), Metro North Health, Brisbane, Australia; Departments of Pharmacy and Intensive Care Medicine, Royal Brisbane and Women’s Hospital, Brisbane, Australia; Division of Anaesthesiology Critical Care Emergency and Pain Medicine, Nîmes University Hospital, University of Montpellier, Nîmes, France; The University of Queensland Centre for Clinical Research, University of Queensland, Brisbane, Australia; The University of Queensland Centre for Clinical Research, University of Queensland, Brisbane, Australia; Infection Management Services, Princess Alexandra Hospital, Metro South Health, Brisbane, Australia; Medical Directorate, NHS England (Midlands), Birmingham, UK; Pharmacy Department, Nottingham University Hospitals NHS Trust, Nottingham, UK; Department of Pharmacy/Infection, Imperial College Healthcare NHS Trust, London, UK; Department of Infectious Diseases, Imperial College London, London, UK; Pharmacy Department, University Hospitals Plymouth NHS Trust, Plymouth, UK; Pharmacy Department, University Hospitals Bristol and Weston NHS Trust, Bristol, UK; Department of Infectious Diseases, Queen Elizabeth University Hospital, Glasgow, UK; British Society for Antimicrobial Chemotherapy, Birmingham, UK; The University of Queensland Centre for Clinical Research, University of Queensland, Brisbane, Australia

## Abstract

**Objectives:**

To evaluate the stability of ceftazidime/avibactam in elastomeric infusers, utilizing the UK’s Yellow Cover Document (YCD) stability testing framework, in conditions representative of OPAT practice.

**Methods:**

Ceftazidime/avibactam was reconstituted with sodium chloride 0.9% (w/v) in two elastomeric infusers at concentrations (dose) levels of 1500/375, 3000/750 and 6000 mg/1500 mg in 240 mL. The infusers were exposed to a fridge storage (2°C–8°C) for 14 days followed by 24 h in-use temperature (32°C).

**Results:**

After 14 days of fridge storage and subsequent 24 h exposure to 32°C, mean ± SD of ceftazidime percent remaining was 75.5% ± 1.8%, 79.9% ± 1.1%, 82.4% ± 0.6%, for Easypump, and 81.7% ± 1.2%, 82.5% ± 0.5%, 85.4% ± 1.1% for Dosi-Fuser devices at the high, intermediate and low doses tested, respectively. For avibactam, mean ± SD percent remaining was 83.2% ± 1.8%, 87.4% ± 2.0%, 93.1% ± 0.9% for Easypump, and 85.1% ± 2.0%, 86.7% ± 0.1%, 92.5% ± 0.1% for Dosi-Fuser devices. The cumulative amount of pyridine generated in the devices ranged from 10.4 mg at low dose to 76.9 mg at high dose. Regression-based simulation showed that the degradation of both ceftazidime and avibactam was <10% for at least 12 h of the running phase, if stored in a fridge for not more than 72 h prior to in-use temperature exposure.

**Conclusions:**

Whilst not meeting the strict UK YCD criteria for ≤5% degradation, ceftazidime/avibactam may be acceptable to administer as a continuous 12 hourly infusion in those territories where degradation of ≤10% is deemed acceptable.

## Introduction

Ceftazidime/avibactam is a novel cephalosporin and β-lactamase inhibitor combination approved for the treatment of complicated urinary tract infections (cUTIs), (including pyelonephritis), complicated intra-abdominal infections (cIAIs), and hospital-acquired pneumonia (HAP) [including ventilator-associated pneumonia (VAP)].^1^ It has a broad spectrum of activity, including MDR bacteria,^2^ which makes this combination an attractive option for treatment of severe infections that require prolonged IV antibiotic therapy, including in outpatient parenteral antimicrobial therapy (OPAT) or hospital-in-the-home (HITH) programmes.^3^

Given the novelty of ceftazidime/avibactam, there have been very few studies to establish its suitability for use in OPAT settings. One case study described a successful treatment of persistent bloodstream infection caused by MDR *Klebsiella pneumoniae* in a HITH service.^4^ Another retrospective cohort study (10 patients) demonstrated the utility of continuous infusion of ceftazidime/avibactam in OPAT, with a twice-daily dosing regimen.^3^ Nevertheless, the potential expanded use of ceftazidime/avibactam in OPAT/HITH programmes is limited by the lack of stability data under the conditions the antibiotic solution would be exposed to in an ambulatory setting. There is particular concern with regard to accumulation of degradation products, particularly pyridine. An excellent review of existing stability studies for ceftazidime (not including avibactam) was recently published by Perks *et al*.^5^ The authors concluded that none of the available studies met regulatory requirements for OPAT use. Additionally, no published data are available in the product information for ceftazidime/avibactam, which describes the drug combination’s sequential stability in refrigerated conditions as well as subsequent continuous 24 h infusion at in-use temperatures. The product information and pharmacopeial assessments currently provide stability-indicating data that do not fulfil requirements as set out in the UKs NHS standard protocol for deriving and assessment of stability of aseptically preparations of small molecules, commonly referred to as the Yellow Cover Document (YCD), which is the only international guideline that specifies testing requirements for stability of drugs used in OPAT/HITH programmes.^6^

A YCD-compliant study by Jamieson *et al*.^7^ tested ceftazidime stability (without avibactam) in elastomeric infusers when reconstituted with sodium chloride 0.9% (w/v). The results did not support 24 h continuous infusion of ceftazidime when considering the YCD acceptance criteria (95%–105% of the study compound present at the end of the testing period). Furthermore, pyridine concentrations were within the British Pharmacopoeia (BP) limit of not more than 0.5% (w/w), only when delivering half (120 mL) of the total volume of the infuser (240 mL). The authors suggested that giving two 12 h infusions per day is possible for ceftazidime solution in sodium chloride 0.9% (w/v). However, there are still no YCD-compliant data supporting the preferred once-daily 24 h continuous-infusion OPAT delivery or the stability of avibactam for 12 hourly dosing with ceftazidime.

The aim of this work was to evaluate the stability of both ceftazidime and avibactam in two different elastomeric infusers, utilizing the UK NHS YCD as a framework, and including the quantification of pyridine, which is the most important degradation product of ceftazidime because of known safety concerns.

## Materials and methods

The acetonitrile solvent used was HPLC grade, Acetonitrile HPLC LiChrosolv (Merck, Darmstadt, Germany). Sodium dihydrogen orthophosphate, analytical reagent (AR) grade, was from Merck (Darmstadt, Germany). Ultrapure water was obtained from a Milli-Q Direct water purification system and ceftazidime/avibactam powder for injection (Zavicefta 2 g/0.5 g, Batch: 2003E2) from Pfizer (Sydney, Australia; Lot #2003E2) was used for calibrators, quality control samples (QCs) and preparation of test solutions for infusers. Pyridine (99.5% purity) was obtained from Sigma–Aldrich (Buchs, Switzerland). Two elastomeric infusion devices from different manufacturers were used: Easypump II LT 270-27-S (B. Braun Ltd, Sheffield, UK; Lot #19E29GE221) and Dosi-fuser L25915-250D1 (Spirit Medical Ltd, Derbyshire, UK; Lot #220070L)

### Assay method

A stability-indicating assay method was developed and validated for simultaneous quantification of ceftazidime, avibactam and pyridine.

#### Chromatographic apparatus and conditions

The method was developed with a Nexera X2 UHPLC system comprising two LC-30AD pumps with degassers, SIL-30AC autosampler, CTO-30AD oven and SPD-M30A photo diode-array detector, controlled by LabSolutions software (Shimadzu Corp., Kyoto, Japan).

The stationary phase was a Symmetry C18 (2.1 × 100 mm, 3.5 µm) analytical column (Waters, Milford, USA) preceded by a Symmetry C18 guard column (2.1 × 10 mm, 3.5 µm) (Waters). Mobile phase was 20 mM sodium phosphate buffer at pH 3.0 in 12.5% acetonitrile delivered isocratically at 0.25 mL/min. The autosampler was held at 4°C, and injection volume was 0.5 µL. The photodiode-array detector scanned from 200 to 500 nm and ceftazidime, avibactam and pyridine were quantified at wavelengths of 260, 230 and 254 nm, respectively.

#### Solutions for analysis

The ceftazidime/avibactam calibrators were prepared in sodium chloride 0.9% (w/v) at concentrations of 0.1/0.025, 0.2/0.05, 0.3/0.075, 0.5/0.125, 1/0.25, 2/0.5 and 3/0.75 mg/mL. Calibration standards for pyridine were prepared in sodium chloride 0.9% (w/v) at concentrations of 0.0015, 0.0075, 0.01, 0.015, 0.075, 0.1 and 0.15 mg/mL. QCs were prepared at concentrations of 25/6.25, 12.5/3.125 and 6.25/1.56 mg/mL ceftazidime/avibactam, respectively, and 0.12, 0.05 and 0.0045 mg/mL pyridine. Aliquots were stored at −80°C until analysis.

In preparation for assay, QCs and samples were diluted with sodium chloride 0.9% (w/v) with a dilution factor of 10 or 5 depending on the concentration of the samples. Diluted samples and QCs were then injected with a set of calibrators.

### Validation of the HPLC method

A quadratic calibration curve was generated from the ceftazidime, avibactam and pyridine peak area with a 1/(concentration)^2^ weighting. Detector linearity was demonstrated from 0.1 to 3 mg/mL for ceftazidime, 0.025 to 0.75 mg/mL for avibactam and 0.0015 to 0.15 mg/mL for pyridine, with *r*^2^ values of 0.9998, 0.9986 and 0.9999, and slopes of 5162515, 433257 and 5896650 for ceftazidime, avibactam and pyridine, respectively. The precision and accuracy of the assay were assessed from six-replicate analysis of QCs at all concentrations across six batches. Precision for ceftazidime, avibactam and pyridine were 1.9%, 1.9% and 1.7%, and accuracy was 6.2%, 4.4% and 2.5%, respectively.

Forced degradation was undertaken using the following stress conditions; 0.1 M hydrochloric acid, 0.1 M sodium hydroxide, 3% hydrogen peroxide, incubation at 50°C and water for acid hydrolysis, base hydrolysis, oxidative stress, heat and control, respectively. Ceftazidime/avibactam was tested at 10/2.5 mg/mL at room temperature and at 50°C with sampling at 0, 60, 150, 210, 270 and 660 min to ascertain the concentration of ceftazidime, avibactam and pyridine.

### Preparation of antibiotic-filled infuser devices

Ceftazidime/avibactam powder for injection was reconstituted with sodium chloride 0.9% (w/v). The contents of the required number of vials were dissolved in a small volume (∼10 mL) of sodium chloride 0.9% (w/v), then transferred into a sterile measuring cylinder in a Class II biosafety cabinet, and subsequently diluted to volume with sodium chloride 0.9% (w/v) to make a centralized stock solution at the desired concentration. Three concentration (dose) levels of ceftazidime/avibactam were tested to cover the range of concentrations of ceftazidime/avibactam likely encountered in clinical practice when administering the total daily dose as continuous 24 h infusion. These include low daily dose (1500 mg/375 mg ceftazidime/avibactam in 240 mL), intermediate daily dose (3000 mg/750 mg ceftazidime/avibactam in 240 mL) and high daily dose (6000 mg/1500 mg ceftazidime/avibactam in 240 mL). The nominal reservoir fill volume of 240 mL was transferred to each of the devices from the central stock using a sterile 60 mL syringe. Devices were prepared in triplicate at each concentration. Flow restrictors and in-line filters were removed from all devices to enable sampling; the outflow line was clamped.

Ceftazidime/avibactam-filled devices were stored in a refrigerator (2°C–8°C) for 14 days without exposure to UV light. Each device was wrapped with aluminium foil to completely cover its surfaces and prevent exposure to light during storage and sampling. Following the 14 day refrigeration, the devices were stored in an incubator at the maximum expected in-use temperature of 32°C for 24 h. Duplicate samples were collected from each individual device for the two device types that were tested at three concentrations in triplicate devices. Samples were collected at 12 different timepoints at 0, 24, 48, 96, 168, 240 and 336 h under refrigeration and at 340, 344, 348, 356 and 360 h at 32°C. The whole study was run in one batch. A total of 432 samples were collected and an aliquot of 0.5 mL of each was immediately stored in a −80°C freezer for concentration measurement.

Samples collected from each device were assessed visually for changes in colour, clarity and any precipitation at similar timepoints of sampling for concentration measurement. The power of hydrogen (pH) of these samples was measured using a Horiba Scientific LAQUAtwin-pH-22 compact pH meter. The presence of subvisible insoluble particles that may be formed due to degradation of the antibiotic within the infusion devices were assessed with the Light Obscuration Particle Count Test using a Beckman Coulter HIAC 9703+ Liquid Particle Counter (Beckman Coulter Inc, CA, USA) in accordance with United States Pharmacopoeia (USP) <787>, <788> and European Pharmacopoeia (EP) 2.9.19. HIAC analysis was performed at time zero, after 14 days of fridge storage, after 12 and 24 h of the running phase at 32°C.

### Data analysis

Statistical software R version 4.2.0 (R Foundation for Statistical Computing, Vienna, Austria) and Microsoft Excel Version 2305 (Microsoft, CA, USA) were used for data manipulation, analysis and presentation. GraphPad Prism version 10.1.2 (GraphPad Software LLC, MA, USA) was used for generation of selected plots.

The cumulative amount of pyridine that would be infused by each device (i.e. delivered to the patient during the running phase) was estimated considering the flow rate of each device. Equations 1, 2 and 3 were used to calculate the cumulative amount delivered over 24 h infusion.


(1) $$\overset{n}{\underset{i=1}{\sum }}\;C_{i}\times V_{i}$$

(2) $$C_{i}=a\times t_{i}+b$$

(3) $$V_{i}=V_{i-1}-f\times (t_{i}-t_{i-1})$$

where: C_i_ is the regression model-predicted concentration of pyridine at time t_i_ post initiation of the running phase; t_i_ is the time elapsed post initiation of the running phase (calculations were made by 1 min increments, where i ranged from 1 min to *n* = 1440 min), V_i_ is the volume of ceftazidime/avibactam remaining within the infusers; f is the flow rate of the device; and a and b are the regression coefficients relating concentration to time.

## Results

### Colour, clarity and precipitation

No visible precipitation was observed for any samples from either device during fridge storage and in-use temperature. Similarly, all samples appeared clear with no visible turbidity. All samples appeared colourless during fridge storage; however, at the in-use temperature, samples taken from devices filled with intermediate and high doses had a faint yellowish appearance. All the devices filled with the low dose maintained a colourless appearance throughout.

### Subvisible liquid particles

All samples scanned for subvisible liquid particle analysis were within the USP <788> subvisible particle testing requirements;^8^ actual counts are summarized in Tables S1 and S2 (available as Supplementary data at *JAC-AMR* Online).

### pH changes

In the Easypump device, the pH significantly increased from baseline with 48 h of fridge storage, and thereafter remain largely stable throughout, regardless of the change from refrigeration to the higher in-use temperature. A similar trend was noted for the Dosi-Fuser device, although the rate and extent of increase were relatively lower in the initial phase until it stabilized after 96 h of fridge storage. Details of the pH data are summarized in Tables S3 and S4.

### Ceftazidime degradation

**Figure 1.**
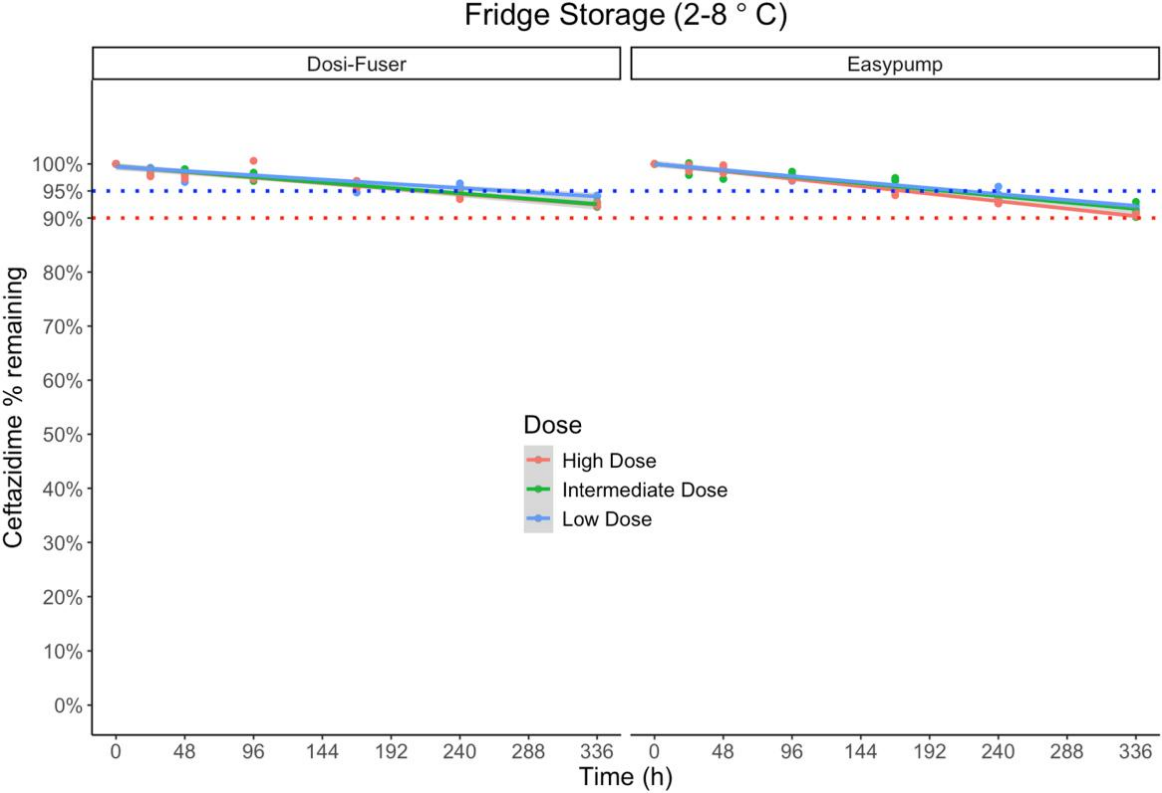
Percentage of ceftazidime remaining during fridge storage (storage 2°C–8°C) for 14 days (336 h) by device and dose. Low dose (1500 mg in 240 mL), intermediate dose (3000 mg in 240 mL) and high dose (6000 mg in 240 mL). The dotted lines indicates the ‘95% active pharmaceutical ingredient remaining’ stability requirement in the UK, and the ‘90% active pharmaceutical ingredient remaining’ stability requirement in other jurisdictions.

**Figure 2.**
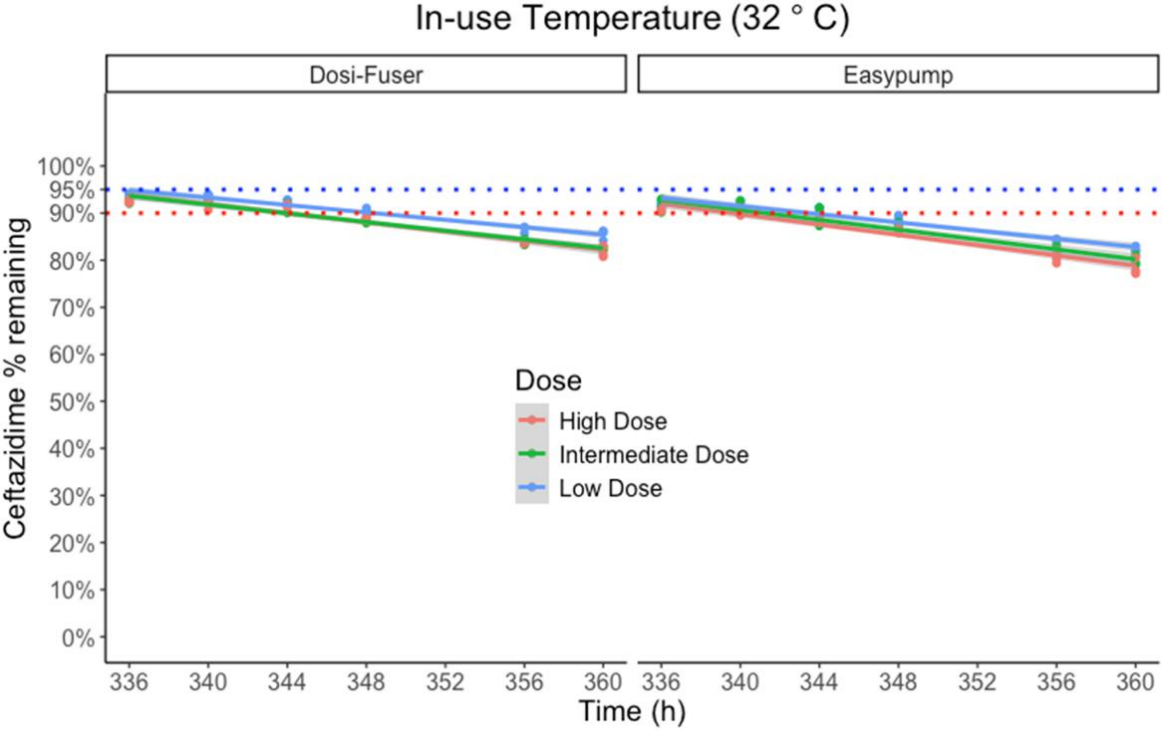
Percentage of ceftazidime remaining during exposure to in-use temperature of 32°C following 14 days (336 h) fridge storage, by device and dose. Low dose (1500 mg in 240 mL), intermediate dose (3000 mg in 240 mL) and high dose (6000 mg in 240 mL). The dotted lines indicates the ‘95% active pharmaceutical ingredient remaining’ stability requirement in the UK, and the ‘90% active pharmaceutical ingredient remaining’ stability requirement in other jurisdictions.

Figures 1 and 2 illustrate the degradation of ceftazidime in sodium chloride 0.9% (w/v) in elastomeric devices over time during fridge storage (2°C–8°C) and subsequent in-use temperature exposure (32°C), respectively. The percentage of degradation remained less than 5% only for 7 days of fridge storage at the high and intermediate concentration, and for 10 days at the low concentration. However, it was less than 10% for all doses at the end of 14 days of fridge storage. At the subsequent exposure to in-use temperature of 32°C, overall degradation of ceftazidime remained ≤10% for only 4–8 h at high and intermediate dose, and 8–12 h at the low dose. The mean ± standard deviation (SD) percentage of ceftazidime remaining at each timepoint of measurement is presented in Table S5.

**Figure 3.**
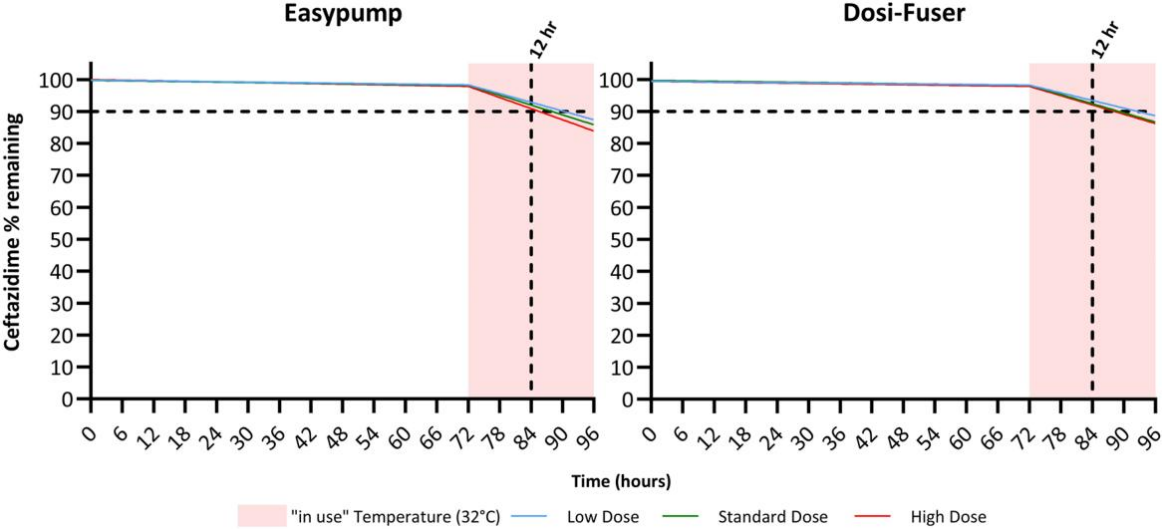
Regression model-simulated percentage of ceftazidime remaining during 72 h fridge storage followed by 24 h exposure to in-use temperature of 32°C; 12 h mark is indicated by the vertical dashed black line from the *x*-axis. The dashed black horizontal line from the *y*-axis indicates the acceptance criteria limit of ‘90% active pharmaceutical ingredient remaining’ used in most jurisdictions.

Figure 3 presents the regression model-simulated percentage of ceftazidime remaining for 72 h, followed by 24 h exposure to in-use temperature of 32°C. In this scenario, the overall degradation during the in-use or ‘running’ phase remained ≤10% for 12 h.

### Avibactam degradation

**Figure 4.**
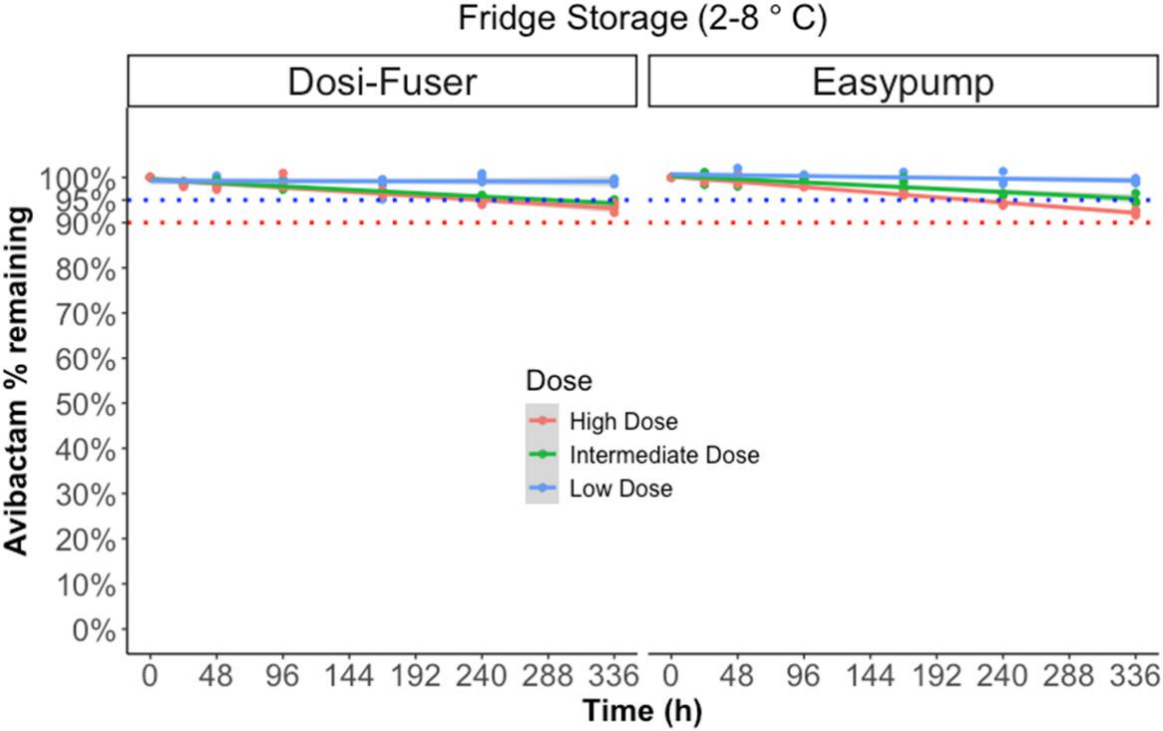
Percentage of avibactam remaining during fridge storage (storage 2°C–8°C) for 14 days (336 h) by device and dose. Low dose (375 mg in 240 mL), intermediate dose (750 mg in 240 mL) and high dose (1500 mg in 240 mL). The dotted lines indicates ‘95% active pharmaceutical ingredient remaining’ stability requirement in the UK, and the ‘90% active pharmaceutical ingredient remaining’ stability requirement in other jurisdictions.

**Figure 5.**
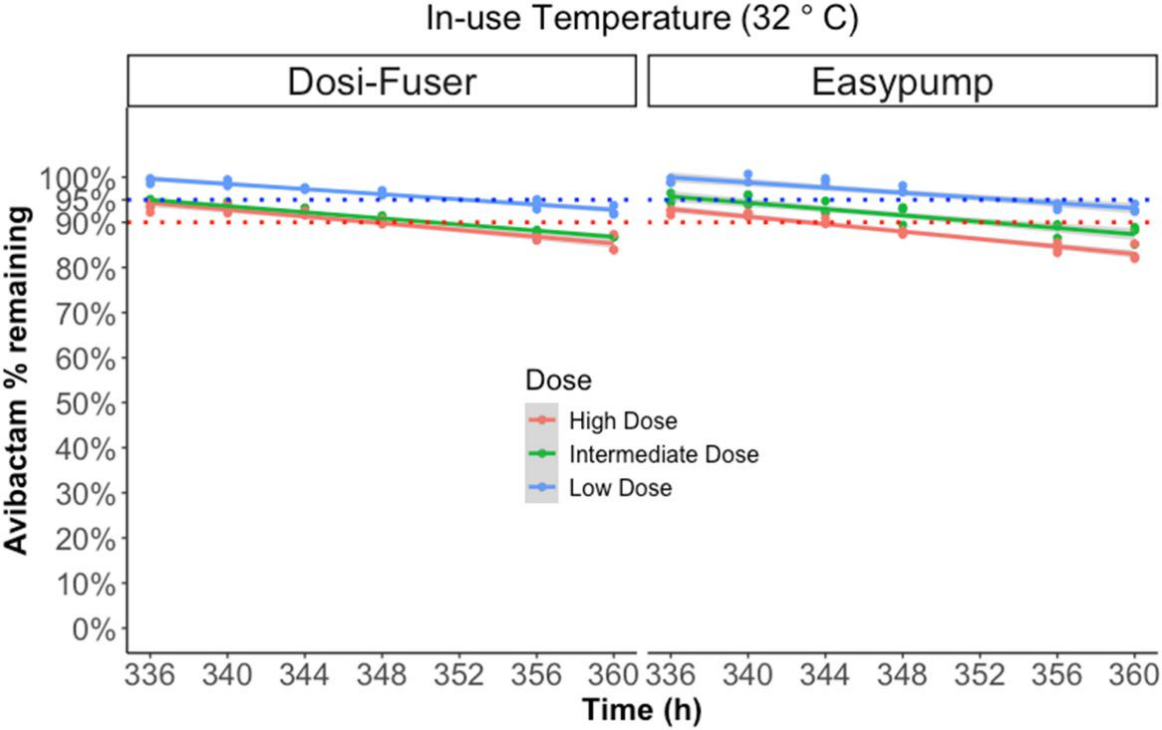
Percentage of avibactam remaining during exposure to in-use temperature of 32°C following 14 days (336 h) fridge storage, by device and dose. Low dose (375 mg in 240 mL), intermediate dose (750 mg in 240 mL) and high dose (1500 mg in 240 mL). The dotted lines indicates ‘95% active pharmaceutical ingredient remaining’ stability requirement in the UK, and the ‘90% active pharmaceutical ingredient remaining’ stability requirement in other jurisdictions.

Figures 4 and 5 illustrate the degradation of avibactam in sodium chloride 0.9% (w/v) in elastomeric devices over time during fridge storage (2°C–8°C) and subsequent in-use temperature exposure (32°C), respectively. The mean percentage of degradation remained less than 5% for 14 days of fridge storage at the low concentration and at intermediate concentration in the Easypump device. At high doses in both devices and the intermediate dose in the Dosi-Fuser device, percent degradation remained less than 5% for up to 10 days of fridge storage. However, it was less than 10% for all doses at the end of 14 days of fridge storage. At the subsequent exposure to in-use temperature of 32°C, overall degradation of avibactam remained ≤10% for 24 h only at low dose; at the high and intermediate dose, ≤10% degradation was noted up to 12 h. The mean ± SD percentage of avibactam remaining at each timepoint of measurement is presented in Table S6.

**Figure 6.**
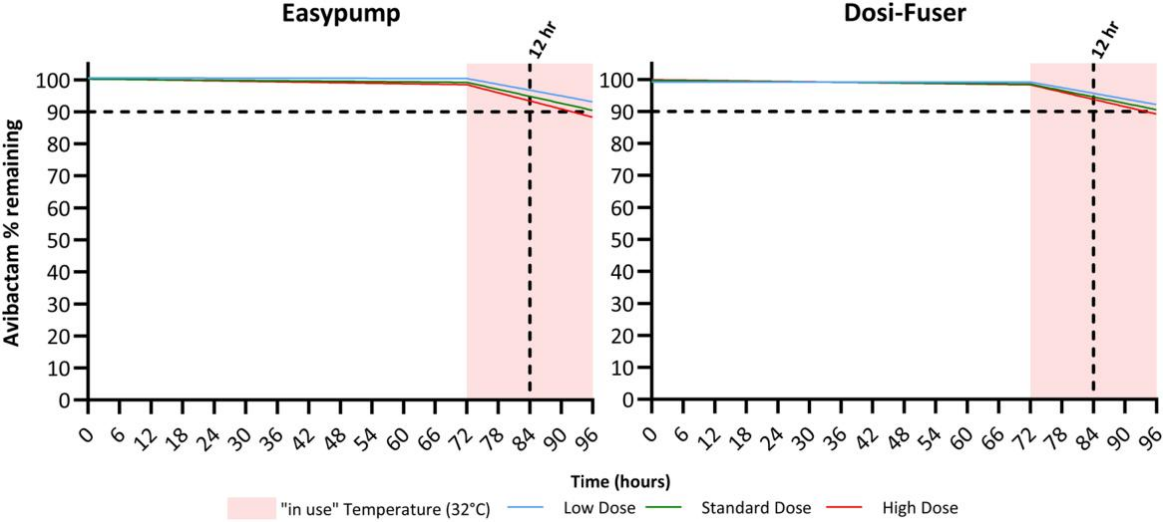
Regression model-simulated percentage of avibactam remaining during 72 h fridge storage followed by 24 h exposure to in-use temperature of 32°C. The 12 h mark is indicated by the vertical dashed black line from the *x*-axis. The dashed black horizontal line from the *y*-axis indicates the acceptance criteria limit of ‘90% active pharmaceutical ingredient remaining’ used in most jurisdictions.

Figures 6 presents the regression model-simulated percentage of avibactam remaining for 72 h followed by 24 h exposure to in-use temperature of 32°C. In this scenario, the overall degradation during the in-use or ‘running phase’ remained ≤10% for over 12 h.

### Pyridine formation

The progressive accumulation of pyridine during 14 days of fridge storage and 24 h in-use temperature exposure is illustrated in Figures S1–S4 for each of the two elastomeric devices studied. The amount of pyridine within the infusers was less than 0.5% (w/w) (BP and EP limit for ceftazidime formulations) of the ceftazidime amount for 7 days of fridge storage at the high and intermediate concentration and for up to 10 days at the low concentration. There was a steep increase in the percentage of pyridine generated during exposure to in-use temperature of 32°C. However, the maximum concentration of pyridine remained far below the 1.1 mg/mL former maximum limit for ceftazidime solutions in the USP (Figures S2 and S4).^9^

**Figure 7.**
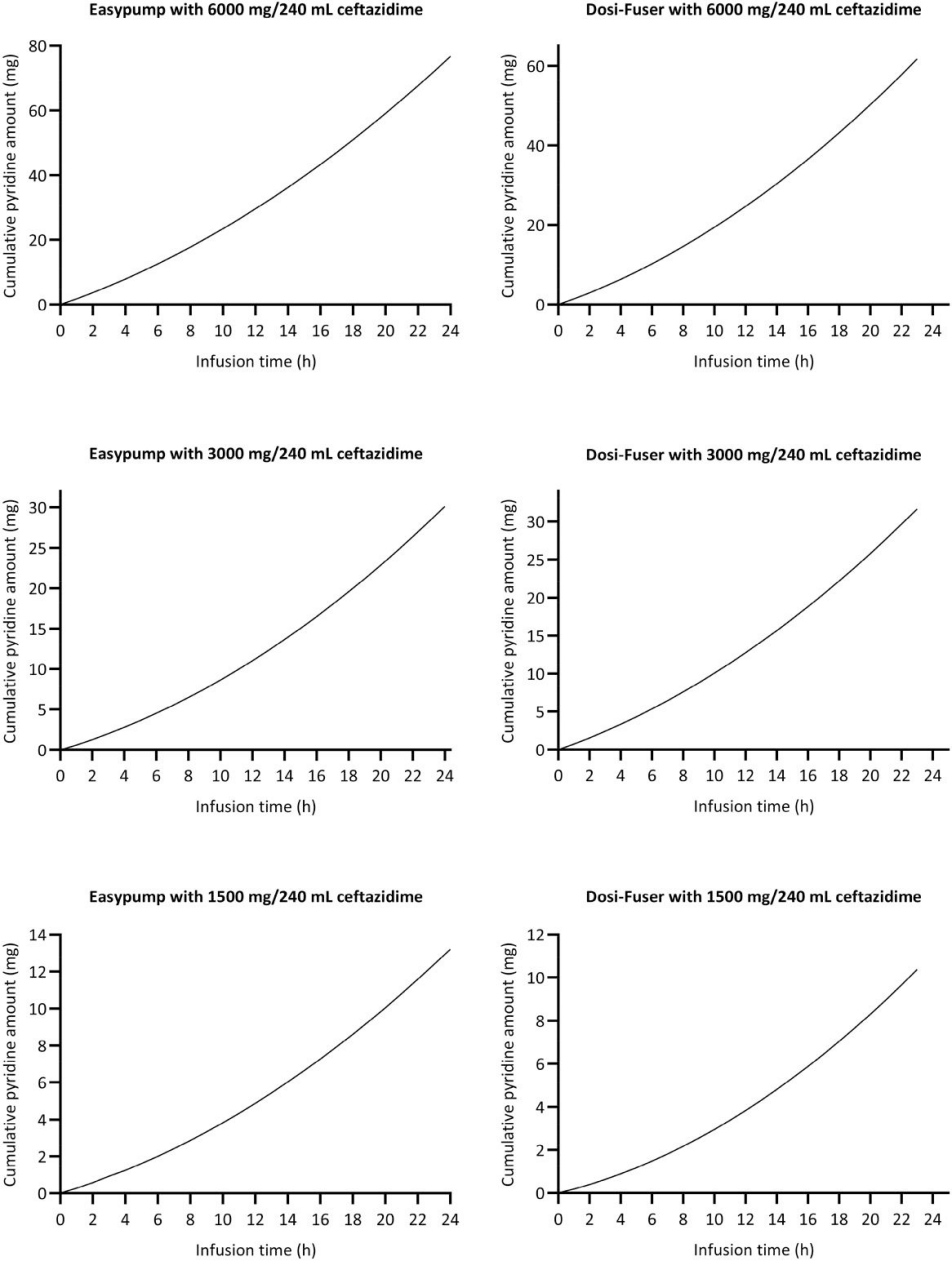
Predicted amount of pyridine that would be delivered to patients during continuous infusion of ceftazidime over 24 h using elastomeric infusion devices.

Even following a short 1–3 day fridge storage and subsequent in-use temperature exposure (running phase), pyridine accumulated above the 0.5% (w/w) limit for formulations within 6–7 h of the ‘running phase’ (Figures S5–S7). For the Easypump device, the estimated cumulative pyridine that would be delivered from 24 h continuous infusion was 76.9, 30.2 and 13.2 mg for the high, intermediate and low doses evaluated, respectively. Similarly, for the Dosi-Fuser device, the cumulative amount of pyridine was 62.0, 31.8 and 10.4 mg, for the high, intermediate and low doses evaluated, respectively. Figure 7 illustrates the cumulative pyridine amount that would be delivered to the patient during continuous infusion of ceftazidime over 24 h.

## Discussion

In this study, we evaluated the sequential stability of ceftazidime/avibactam in two elastomeric devices when stored under refrigeration, followed by exposure to in-use temperature (32°C), and quantified maximal amount of pyridine that could be passed to patients from degradation of ceftazidime. As far as we know, there is no other published YCD-compliant stability study for ceftazidime/avibactam, although a few previous studies have evaluated either ceftazidime alone^5,10^ or with avibactam^11^ without meeting recommended testing conditions applicable to OPAT. The main findings of this study are: (i) avibactam has a better stability than ceftazidime both during fridge storage and in-use temperature exposure (32°C); (ii) the overall sequential stability following fridge storage plus in-use exposure at 32°C does not support 24 h continuous infusion of ceftazidime/avibactam; (iii) the results do support 12 h continuous infusion (twice-daily dosing) in regions where the acceptable limit of degradation is 10% such as the USA; however not in regions that follow the 5% degradation limit of the UK NHS YCD; (iv) ceftazidime/avibactam-filled devices should not be stored in a fridge for more than 72 h prior to the running phase to enable 12 h twice-daily continuous infusion; and finally (v) the maximum amount of cumulative pyridine generated from ceftazidime degradation that would be passed to patients over 24 h infusion was less than 100 mg.^12^

Only one study by Jamieson *et al*.^7^ was able to demonstrate alignment with the contemporary YCD stability testing guidelines for OPAT use.^7^ Under comparable test conditions, Jamieson *et al.*^7^ found ceftazidime stability results consistent with ours, recommending 12 hourly infusion of ceftazidime following 48 h fridge storage, although they have not studied avibactam. Also consistent with our results, they found that the monograph limits of pyridine in ceftazidime formulation (0.5% w/w) are often reached quickly before significant loss of ceftazidime (Figures S1 and S2).

Pyridine is one of the four major monograph-specified impurities associated with ceftazidime formulations and is of particular interest due to its potential toxicities. Structurally, ceftazidime contains a core pyridine ring, which is usually released during hydrolytic degradation in aqueous solutions.^13^ Much of what is known about the potential toxicity of pyridine to humans is from its failed development as a potential drug for epilepsy nearly 100 years ago. Pollock *et al.*^14^ administered pyridine to five patients suffering from epilepsy; although at low doses, no toxicity was observed. Dose escalation (1.85 to 2.46 mL of pyridine per day; ∼2 g/day) resulted in marked toxicities manifesting as hepatorenal disease with uraemic symptoms, subconjunctival haemorrhages, oedema, jaundice and delirium, leading to fatality in one of the patients. The acute lethal or toxic dose of pyridine for humans is largely unknown; 500 mg/kg has been suggested as the lowest lethal dose (LDL_0_) in the literature.^15^ In a rodent toxicity study, the no-observed-adverse-effect-level (NOAEL) was 1 mg/kg/day.^16^ Nevertheless, there has been no documented report of pyridine toxicity or fatality in humans from administration of ceftazidime.^12^

Considering the overall experience that pyridine-related toxicities have not been documented over decades of ceftazidime use, and their observation that generally less than 100 mg of pyridine is produced when doses of ceftazidime are ≤6 g/day, Jones *et al*.^12^ suggested a 100 mg limit as a ‘realistic standard against which acceptability of ceftazidime administration by continuous intravenous infusion should be measured’. Whilst this limit requires scientific validation, in the current study the cumulative daily amount of pyridine that would be delivered by continuous infusion of ceftazidime/avibactam was far less than 100 mg. The maximum estimated amount was 76.9 mg for the high daily dose (6000 mg in 240 mL) of ceftazidime in the Easypump device, and 60.2 mg in the Dosi-Fuser device. These values are comparable to the total delivered amount estimated by Bourget *et al*.^17^ following exposure of a ceftazidime-filled Baxter LV10 elastomeric device to in-use temperature of 33°C (30.4 mg in 6000 mg/115 mL ceftazidime solution given 12 hourly). In our study, at intermediate and low ceftazidime daily doses of 3000 and 1500 mg, the cumulative amount of pyridine would be even less, for example 30.2 and 13.2 mg, respectively, for the Easypump device. In addition, concentrations of pyridine observed in this *in vitro* study will not be achieved *in vivo* due to the slow continuous infusion administration, and the accumulation in patients at the end of infusion will be far lower than the cumulative amount administered as pyridine gets rapidly cleared from the body, predominantly renally.^18^ However, the exact human half-life of pyridine is uncertain and therefore the extent of accumulation after repeated daily doses and its potential impact should be carefully investigated. In addition, interpatient variability in the disposition of pyridine is likely, which may be further complicated by underlying pathophysiological conditions of the patient. For example, the lower total daily dose evaluated in this study (1500 mg of ceftazidime) may be used in the context of renal dysfunction and therefore potential accumulation of pyridine in such cases may be a concern.

The relevance of BP and EP limits for pyridine, 0.5% (w/w) and the 2 mg permitted daily exposure (PDE) for residual pyridine when used as a solvent, have been discussed in detail by Jones *et al*.^12^ In the current USP, for ceftazidime for injection (which is a sterile mixture of ceftazidime and sodium carbonate or arginine), pyridine limits of not more than 0.4% (w/w) where it contains sodium carbonate and not more than 0.3% (w/w) where it contains arginine, are specified. All these limits are unlikely to be met by any ceftazidime solutions in commonly used solvents for intravenous injection. Of note, the amount of pyridine present in products before reconstitution for clinical use may already be close to this limit. On the other hand, the former USP upper limit of 1.1 mg/ml pyridine for pharmaceutically acceptable ceftazidime solutions provided a relatively higher amount of allowable pyridine in ceftazidime solutions,^9,17^ which was met at all doses tested (Figures S3 and S4); in fact, the maximum pyridine concentration measured at the end of the running phase in this study was 0.48 mg/mL at high dose.

In conclusion, ceftazidime/avibactam reconstituted in sodium chloride 0.9% (w/v) at doses (concentrations) up to 6000 mg/1500 mg in 240 mL, does not meet the ≤5% degradation limit in OPAT use conditions as per the UK NHS YCD. However, it is stable for more than 12 h at in-use temperature of 32°C when considering the ≤10% degradation limit acceptable in some jurisdictions. When used in OPAT settings in territories where ≤10% degradation is acceptable, prolonged fridge storage for greater than 72 h should be avoided to keep the overall sequential degradation to less than 10%. However, the amount of pyridine generated from ceftazidime degradation under the conditions of use in OPAT (fridge storage followed by infusion at ambient temperature) does not meet the BP and EP limit of 0.5% (w/w) or the USP limit of 0.4% (w/w) for ceftazidime for injection, and the 2 mg PDE stated by the EMA and the USP for residual pyridine when used as a solvent.

## Supplementary Material

dlae056_Supplementary_Data

## Transparency declarations

J.A.R. is a consultant/advisory board member of: Qpex (2022); Gilead (2022); Advanz Pharma (2022); Pfizer (2020); Sandoz (2020); Wolters Kluwer (2021); MSD (2021); and Summit Pharma (2021). J.A.R. has received funding from: Pfizer (2023); MSD (2022); Gilead (2022); and Cipla (2021) to speak at conferences. J.A.R. received industry grants from: Pfizer (2023); Qpex (2021) and BSAC (2021). M.G. is a consultant/advisory board member of Pfizer (2023) and Menarini (2023). T.H. has received support to attend a conference and advisory board fees from Eumedica (2019) and Pfizer (2017). R.A.S. is a consultant/advisory board member and/or speaker for Advanz, Pfizer, Menarini, Shionogi and Napp. M.G., C.J., T.H., M.S., and R.A.S. are members of the British Society for Antimicrobial Chemotherapy (BSAC) Drug Stability Testing Working Group, part of the OPAT Initiative. M.G. and R.A.S are co-leads of the BSAC OPAT Initiative.

## Supplementary data

Figures S1–S7 and Tables S1–S6 are available as Supplementary data at *JAC-AMR* Online.
