# Supplementary material for: Evaluation of the stability of ceftazidime/avibactam in elastomeric infusion devices used for outpatient parenteral antimicrobial therapy utilizing a national stability protocol framework
Source: JAC Antimicrob Resist. 2024 Apr 5;6(2):dlae056. doi: 10.1093/jacamr/dlae056 (PMC10995722; doi:10.1093/jacamr/dlae056)
Supplement: dlae056_Supplementary_Data [file dlae056_supplementary_data.docx]

**Supplementary File**

Table S1: Subvisible particle cumulative counts per millilitre for particles equal to or greater than 10 and 25 microns in Easypump infuser devices.

| **Storage condition** |  | **Easypump subvisible particles (mean ± SD cc/mL)** | | | | | |
| --- | --- | --- | --- | --- | --- | --- | --- |
|  |  | **Low dose** | | **Intermediate dose** | | **High dose** | |
|  | **Time (hr)** | **10 µm** | **25 µm** | **10 µm** | **25 µm** | **10 µm** | **25 µm** |
| **Refrigeration (4°C)** | **0** | 0.50 ± 0.00 | 0.33 ± 0.00 | 1.50 ± 0.00 | 1.00 ± 0.00 | 2.17 ± 0.00 | 0.83 ± 0.00 |
|  | **336** | 0.33 ± 0.34 | 0.17 ± 0.09 | 0.33 ± 0.34 | 0.33 ± 0.17 | 0.33 ± 0.34 | 0.33 ± 0.25 |
| **"In-Use" (32°C)** | **348** | 0.00 ± 0.00 | 0.00 ± 0.00 | 0.17 ± 0.10 | 0.00 ± 0.00 | 0.83 ± 0.75 | 0.00 ± 0.00 |
|  | **360** | 0.00 ± 0.10 | 0.00 ± 0.00 | 0.00 ± 0.10 | 0.00 ± 0.00 | 0.83 ± 0.17 | 0.00 ± 0.00 |
| cc/mL cumulative counts/millilitre, hr hours | | |  |  |  |  |  |

Table S2: Subvisible particle cumulative counts per millilitre for particles equal to or greater than 10 and 25 microns in Dosi-Fuser infuser devices.

| **Storage condition** |  | **Dosi-Fuser subvisible particles (mean ± SD cc/mL)** | | | | | |
| --- | --- | --- | --- | --- | --- | --- | --- |
|  |  | **Low dose** | | **Intermediate dose** | | **High dose** | |
|  | **Time (hr)** | **10 µm** | **25 µm** | **10 µm** | **25 µm** | **10 µm** | **25 µm** |
| **Refrigeration (4°C)** | **0** | 0.67 ± 0.00 | 0.50 ± 0.00 | 0.83 ± 0.00 | 0.33 ± 0.00 | 2.50 ± 0.00 | 1.33 ± 0.00 |
|  | **336** | 0.00 ± 0.10 | 0.00 ± 0.10 | 0.33 ± 0.34 | 0.17 ± 0.35 | 0.50 ± 0.25 | 0.33 ± 0.29 |
| **"In-Use" (32°C)** | **348** | 0.17 ± 0.17 | 0.00 ± 0.00 | 0.00 ± 0.10 | 0.00 ± 0.00 | 0.17 ± 0.09 | 0.00 ± 0.00 |
|  | **360** | 0.00 ± 0.10 | 0.00 ± 0.00 | 0.83 ± 0.54 | 0.00 ± 0.00 | 0.00 ± 0.00 | 0.00 ± 0.00 |
| cc/mL cumulative counts/millilitre, hr hours | | |  |  |  |  |  |

Table S3 Change in pH of ceftazidime/avibactam solution in Easypump II LT 270-27-S elastomeric devices filled at low, intermediate, and high doses during fridge and in-use temperature storage.

| Temperature condition | | Time | Mean ± SD observed pH and change in mean pH from baseline by dose | | | | | |
| --- | --- | --- | --- | --- | --- | --- | --- | --- |
|  |  |  | Low dose | | Intermediate dose | | High dose | |
|  |  |  | Mean ± SD | Δ pH | Mean ± SD | Δ pH | Mean ± SD | Δ pH |
| Fridge storage (2°C - 8°C) | | 0 | 6.59 ± 0.04 | 0.00 | 6.61 ± 0.07 | 0.00 | 6.49 ± 0.07 | 0.00 |
|  |  | 24 | 7.25 ± 0.06 | -0.67 | 7.12 ± 0.12 | -0.50 | 7.16 ± 0.17 | -0.66 |
|  |  | 48 | 7.43 ± 0.09 | -0.84 | 7.37 ± 0.18 | -0.75 | 7.53 ± 0.11 | -1.04 |
|  |  | 96 | 7.44 ± 0.05 | -0.85 | 7.46 ± 0.09 | -0.85 | 7.54 ± 0.07 | -1.05 |
|  |  | 168 | 7.56 ± 0.07 | -0.97 | 7.49 ± 0.12 | -0.88 | 7.73 ± 0.04 | -1.24 |
|  |  | 240 | 7.41 ± 0.01 | -0.82 | 7.48 ± 0.11 | -0.87 | 7.55 ± 0.07 | -1.06 |
|  |  | 336 | 7.63 ± 0.03 | -1.04 | 7.68 ± 0.06 | -1.07 | 7.83 ± 0.05 | -1.34 |
| In-use temperature (32°C) | | 340 | 7.47 ± 0.04 | -0.89 | 7.51 ± 0.08 | -0.90 | 7.61 ± 0.08 | -1.12 |
|  |  | 344 | 7.47 ± 0.04 | -0.88 | 7.47 ± 0.06 | -0.86 | 7.48 ± 0.08 | -0.99 |
|  |  | 348 | 7.53 ± 0.08 | -0.95 | 7.56 ± 0.11 | -0.94 | 7.57 ± 0.09 | -1.07 |
|  |  | 356 | 7.37 ± 0.1 | -0.78 | 7.42 ± 0.02 | -0.81 | 7.46 ± 0.02 | -0.97 |
|  |  | 360 | 7.49 ± 0.05 | -0.91 | 7.44 ± 0.1 | -0.82 | 7.47 ± 0.08 | -0.98 |
| pH, power of hydrogen; SD, standard deviation. | | | | | | | |  |

Table S4 Change in pH of ceftazidime/avibactam solution in Dosi-Fuser L25915-250D1S elastomeric devices filled at low, intermediate, and high doses during fridge and in-use temperature storage.

| Temperature condition | Time | Mean ± SD observed pH and change in mean pH from baseline by dose | | | | | |
| --- | --- | --- | --- | --- | --- | --- | --- |
|  |  | Low dose | | Intermediate dose | | High dose | |
|  |  | Mean ± SD | Δ pH | Mean ± SD | Δ pH | Mean ± SD | Δ pH |
| Fridge storage (2°C - 8°C) | 0 | 6.62 ± 0.05 | 0.00 | 6.52 ± 0.01 | 0.00 | 6.69 ± 0.06 | 0.00 |
|  | 24 | 6.93 ± 0.1 | -0.31 | 6.96 ± 0.03 | -0.44 | 6.88 ± 0.06 | -0.19 |
|  | 48 | 7.15 ± 0.06 | -0.53 | 7.06 ± 0.03 | -0.54 | 7.1 ± 0.1 | -0.41 |
|  | 96 | 7.14 ± 0.03 | -0.52 | 7.19 ± 0.09 | -0.67 | 7.25 ± 0.05 | -0.56 |
|  | 168 | 7.35 ± 0.03 | -0.73 | 7.34 ± 0.03 | -0.82 | 7.41 ± 0.06 | -0.72 |
|  | 240 | 7.29 ± 0.02 | -0.67 | 7.37 ± 0.02 | -0.86 | 7.37 ± 0.06 | -0.68 |
|  | 336 | 7.53 ± 0.04 | -0.91 | 7.58 ± 0.03 | -1.06 | 7.63 ± 0.03 | -0.94 |
| In-use temperature (32°C) | 340 | 7.36 ± 0.05 | -0.74 | 7.44 ± 0.02 | -0.92 | 7.43 ± 0.06 | -0.74 |
|  | 344 | 7.34 ± 0.12 | -0.72 | 7.34 ± 0.06 | -0.82 | 7.47 ± 0.05 | -0.78 |
|  | 348 | 7.51 ± 0.02 | -0.89 | 7.5 ± 0.02 | -0.99 | 7.5 ± 0.02 | -0.81 |
|  | 356 | 7.41 ± 0.04 | -0.79 | 7.39 ± 0.02 | -0.88 | 7.47 ± 0.05 | -0.78 |
|  | 360 | 7.39 ± 0.04 | -0.77 | 7.41 ± 0.01 | -0.90 | 7.38 ± 0.03 | -0.69 |
| pH, power of hydrogen; SD, standard deviation. | | | | | | | |

Table S5. Percentage of ceftazidime remaining during fridge storage (storage 2 to 8°C) for 14 days (336 h) followed by in-use temperature exposure (32°C) for 24 hours. Low dose (1500 mg/ 240 mL), Intermediate dose (3000 mg in 240 mL) and High dose (6000 mg in 240 mL).

| **Storage condition** |  | **Easypump**  **Ceftazidime % remaining (mean ± SD)** | | | | | | **Dosi-Fuser**  **Ceftazidime % remaining (mean ± SD)** | | | |  |
| --- | --- | --- | --- | --- | --- | --- | --- | --- | --- | --- | --- | --- |
|  | **Time (hr)** | **Low dose** | | **Intermediate dose** | **High dose** | | | **Low dose** | | **Intermediate dose** | **High dose** |  |
| **Refrigeration (2 to 8°C)** | **0** | 100.00 ± 0.00 | | 100.00 ± 0.00 | 100.00 ± 0.00 | | | 100.00 ± 0.00 | | 100.00 ± 0.00 | 100.00 ± 0.00 |  |
|  | **24** | 99.37 ± 0.29 | | 99.33 ± 1.21 | 99.19 ± 0.56 | | | 99.17 ± 0.18 | | 98.51 ± 0.51 | 98.42 ± 0.63 |  |
|  | **48** | 98.90 ± 0.58 | | 98.37 ± 1.00 | 99.14 ± 0.78 | | | 98.11 ± 1.28 | | 98.70 ± 0.44 | 97.67 ± 0.39 |  |
|  | **96** | 97.55 ± 0.63 | | 97.83 ± 0.70 | 97.09 ± 0.10 | | | 98.11 ± 0.12 | | 97.50 ± 0.80 | 98.42 ± 1.86 |  |
|  | **168** | 96.21 ± 0.56 | | 96.54 ± 1.19 | 94.76 ± 0.51 | | | 96.02 ± 1.16 | | 96.02 ± 0.44 | 96.35 ± 0.65 |  |
|  | **240** | 94.72 ± 0.97 | | 93.85 ± 0.30 | 92.91 ± 0.40 | | | 95.94 ± 0.46 | | 94.63 ± 0.47 | 94.00 ± 0.51 |  |
|  | **336** | 92.01 ±0.40 | | 91.47 ± 1.43 | 90.67 ± 0.25 | | | 94.06 ± 0.09 | | 92.58 ± 0.49 | 92.43 ± 0.35 |  |
| **"In-Use" (32°C)** | **340** | 91.75 ± 1.05 | | 91.04 ± 1.50 | 90.02 ± 0.43 | | | 93.69 ± 0.45 | | 92.31 ± 0.33 | 91.95 ± 1.09 |  |
|  | **344** | 90.95 ± 0.42 | | 89.46 ± 1.97 | 88.71 ± 0.50 | | | 92.47 ± 0.61 | | 90.78 ± 1.09 | 91.53 ± 0.31 |  |
|  | **348** | 88.63 ± 0.99 | | 87.51 ± 0.60 | 86.23 ± 0.43 | | | 90.19 ± 1.10 | | 88.63 ± 0.70 | 88.84 ± 0.24 |  |
|  | **356** | 84.17 ± 0.54 | | 81.63 ± 1.17 | 80.39 ± 1.03 | | | 86.59 ± 0.74 | | 83.73 ± 0.68 | 83.64 ± 0.15 |  |
|  | **360** | 82.38 ± 0.55 | | 79.91 ± 1.09 | 78.50 ± 1.78 | | | 85.42 ± 1.10 | | 82.48 ± 0.48 | 81.68 ± 1.20 |  |
| hr, hours; SD, standard deviation | | |  | | |  |  | |  | | |  |

Table S6. Percentage of avibactam remaining during fridge storage (storage 2 to 8°C) for 14 days (336 h) followed by in-use temperature exposure (32°C) for 24 hours. Low dose (1500 mg/ 240 mL), Intermediate dose (3000 mg in 240 mL) and High dose (6000 mg in 240 mL).

|  |  | **Easypump**  **Avibactam % remaining (mean ± SD)** | | | | **Dosi-Fuser**  **Avibactam % remaining (mean ± SD)** | | | | | | |  |  |
| --- | --- | --- | --- | --- | --- | --- | --- | --- | --- | --- | --- | --- | --- | --- |
| **Storage condition** | **Time (hr)** | **Low dose** | **Intermediate dose** | | **High dose** | | **Low dose** | | | **Intermediate dose** | | **High dose** | |  |
| **Refrigeration (2 to 8°C )** | **0** | 100.00 ± 0.00 | 100.00 ± 0.00 | | 100.00 ± 0.00 | | 100.00 ± 0.00 | | | 100.00 ± 0.00 | | 100.00 ± 0.00 | |  |
|  | **24** | 100.68 ± 0.33 | 100.19 ± 1.55 | | 99.33 ± 0.63 | | 98.86 ± 0.19 | | | 98.64 ± 0.41 | | 98.59 ± 0.58 | |  |
|  | **48** | 101.13 ± 0.81 | 99.18 ± 1.12 | | 99.58 ± 0.95 | | 99.29 ± 1.41 | | | 99.00 ± 0.54 | | 97.89 ± 0.49 | |  |
|  | **96** | 100.29 ± 0.34 | 99.31 ± 0.56 | | 97.94 ± 0.03 | | 99.27 ± 0.37 | | | 98.02 ± 0.94 | | 98.98 ± 1.72 | |  |
|  | **168** | 100.27 ± 0.86 | 98.71 ± 1.42 | | 96.27 ± 0.27 | | 97.74 ± 2.28 | | | 96.70 ± 0.50 | | 96.69 ± 0.57 | |  |
|  | **240** | 99.68 ± 1.47 | 96.30 ± 0.28 | | 94.07 ± 0.28 | | 99.97 ± 0.94 | | | 95.94 ± 0.17 | | 94.37 ± 0.43 | |  |
|  | **336** | 99.15 ± 0.57 | 95.19 ± 1.16 | | 92.32 ± 0.63 | | 99.23 ± 0.62 | | | 94.33 ± 0.81 | | 93.31 ± 1.07 | |  |
| **"In-Use" (32°C)** | **340** | 98.50 ± 2.42 | 94.75 ± 1.22 | | 91.82 ± 0.63 | | 98.77 ± 0.70 | | | 94.21 ± 0.30 | | 93.12 ±1.02 | |  |
|  | **344** | 99.01 ± 0.78 | 93.08 ± 1.45 | | 90.15 ± 0.67 | | 97.48 ± 0.26 | | | 92.42 ± 0.70 | | 92.17 ± 0.52 | |  |
|  | **348** | 97.23 ± 0.81 | 91.86 ± 2.14 | | 87.70 ± 0.45 | | 96.50 ± 0.55 | | | 91.03 ± 0.42 | | 90.35 ± 0.65 | |  |
|  | **356** | 93.47 ± 0.72 | 88.39 ± 1.61 | | 84.17 ± 1.17 | | 94.00 ± 1.09 | | | 87.85 ± 0.48 | | 86.42 ± 0.40 | |  |
|  | **360** | 93.09 ± 0.91 | 87.40 ± 2.02 | | 83.23 ± 1.78 | | 92.54 ± 1.09 | | | 86.82 ± 0.07 | | 85.07 ± 1.98 | |  |
| hr, hours; SD, standard deviation | | | |  |  | | |  |  | |  | | | |


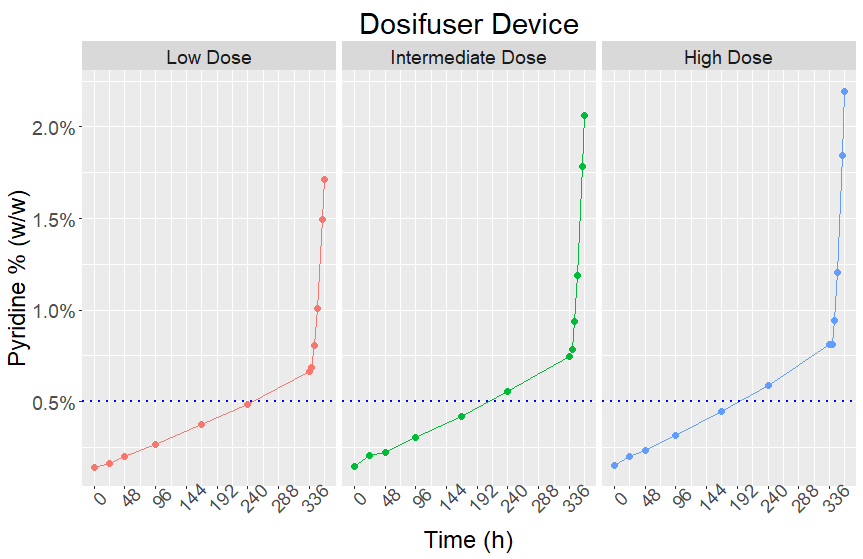


**Figure S1**. The percentage of pyridine relative to baseline ceftazidime amount *(%w/w)* during 14 days of fridge storage followed by 24 hours exposure to in-use temperature (32°C) in Dosi-Fuser L25915-250D1 device. The blue dotted line indicates the allowable limit per European and British Pharmacopoeias.


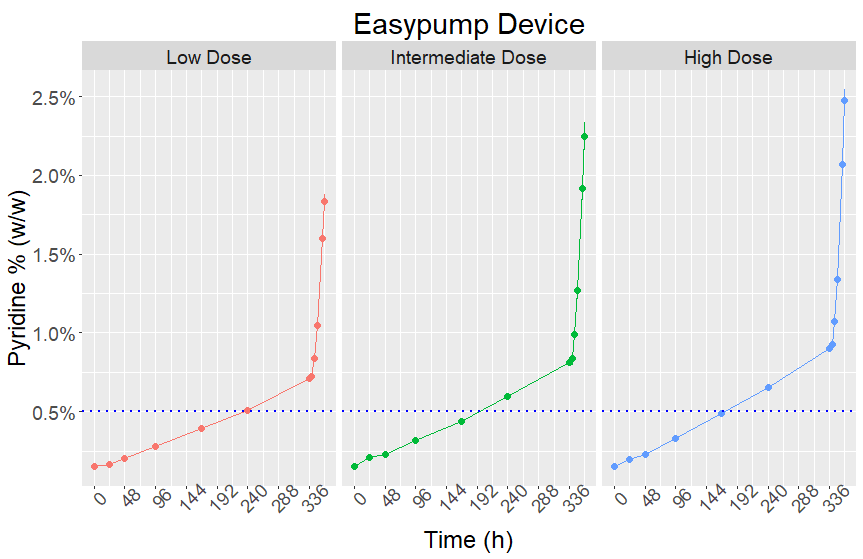


**Figure S2**. The percentage of pyridine relative to baseline ceftazidime amount *(%w/w)* during 14 days of fridge storage followed by 24 hours exposure to in-use temperature (32°C) in Easypump II LT 270-27-S device. The blue dotted line indicates the allowable limit per European and British Pharmacopoeias.


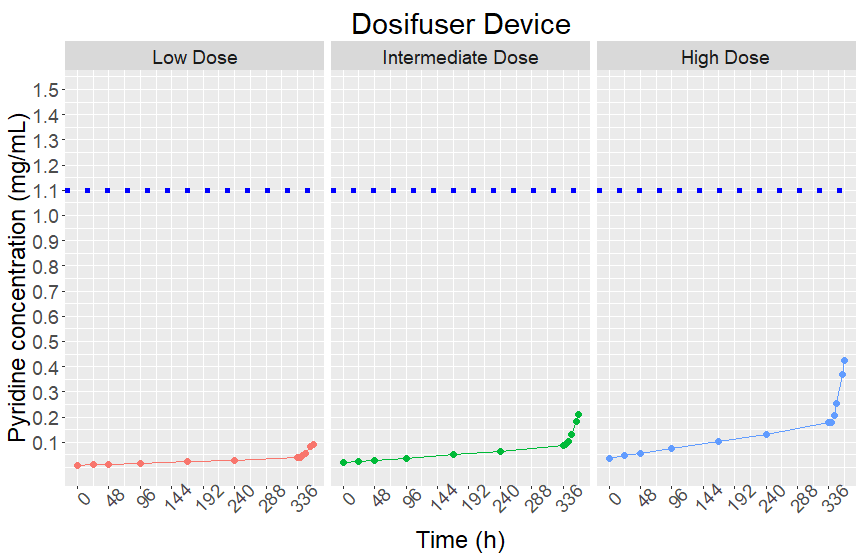


**Figure S3**. The concentration of pyridine (mg/mL) during 14 days of fridge storage followed by 24 hours exposure to in-use temperature (32°C) in Dosi-Fuser L25915-250D1 device.


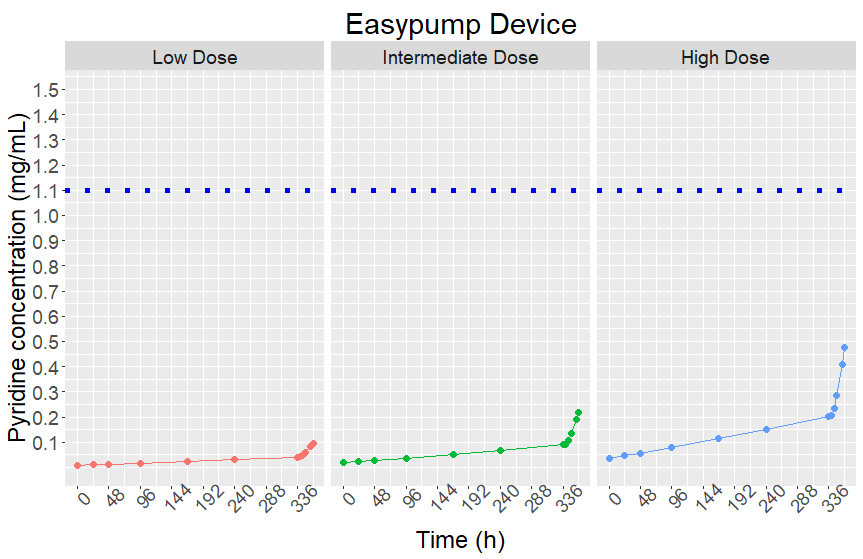


**Figure S4**. The concentration of pyridine (mg/mL) during 14 days of fridge storage followed by 24 hours exposure to in-use temperature (32°C) in Easypump II LT 270-27-S device.


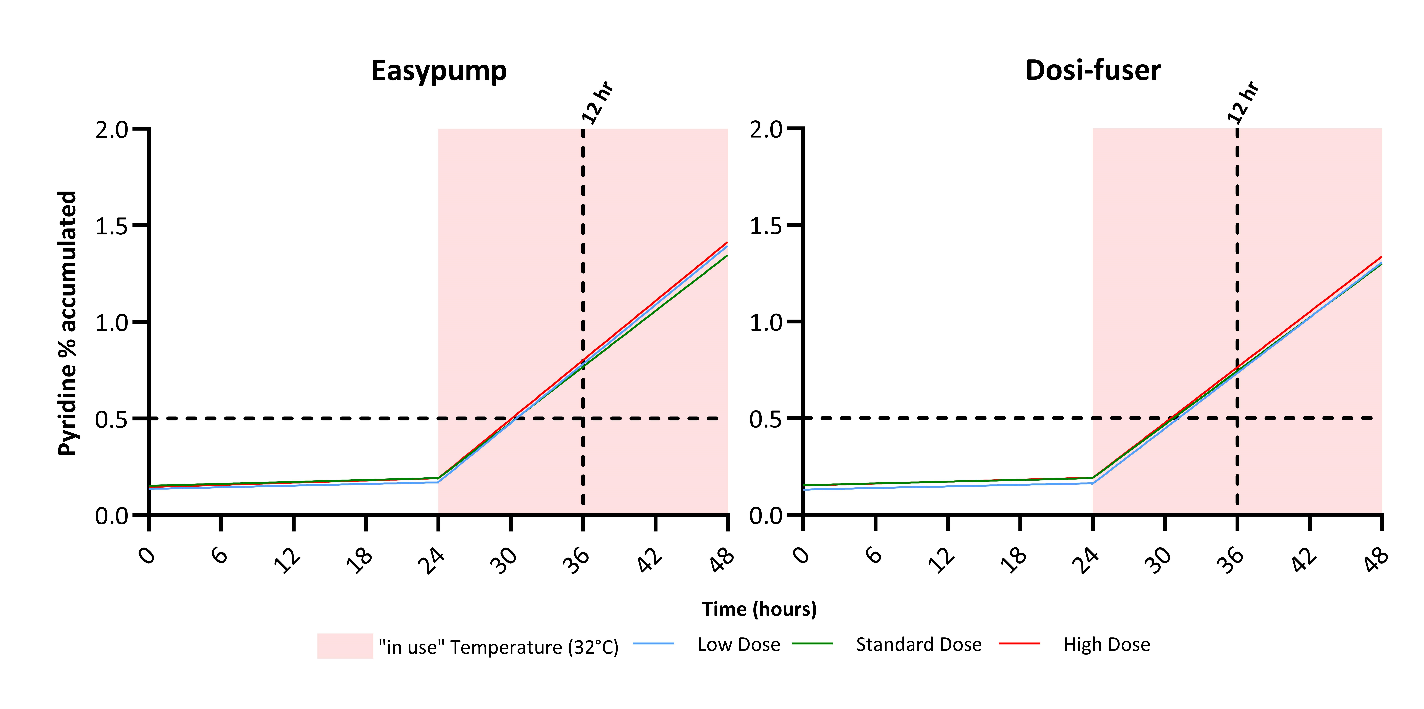


**Figure S5**. Regression model simulated-percentage of pyridine relative to baseline ceftazidime amount *(%w/w)* during 24 hours of fridge storage followed by 24 hours exposure to in-use temperature (32°C).


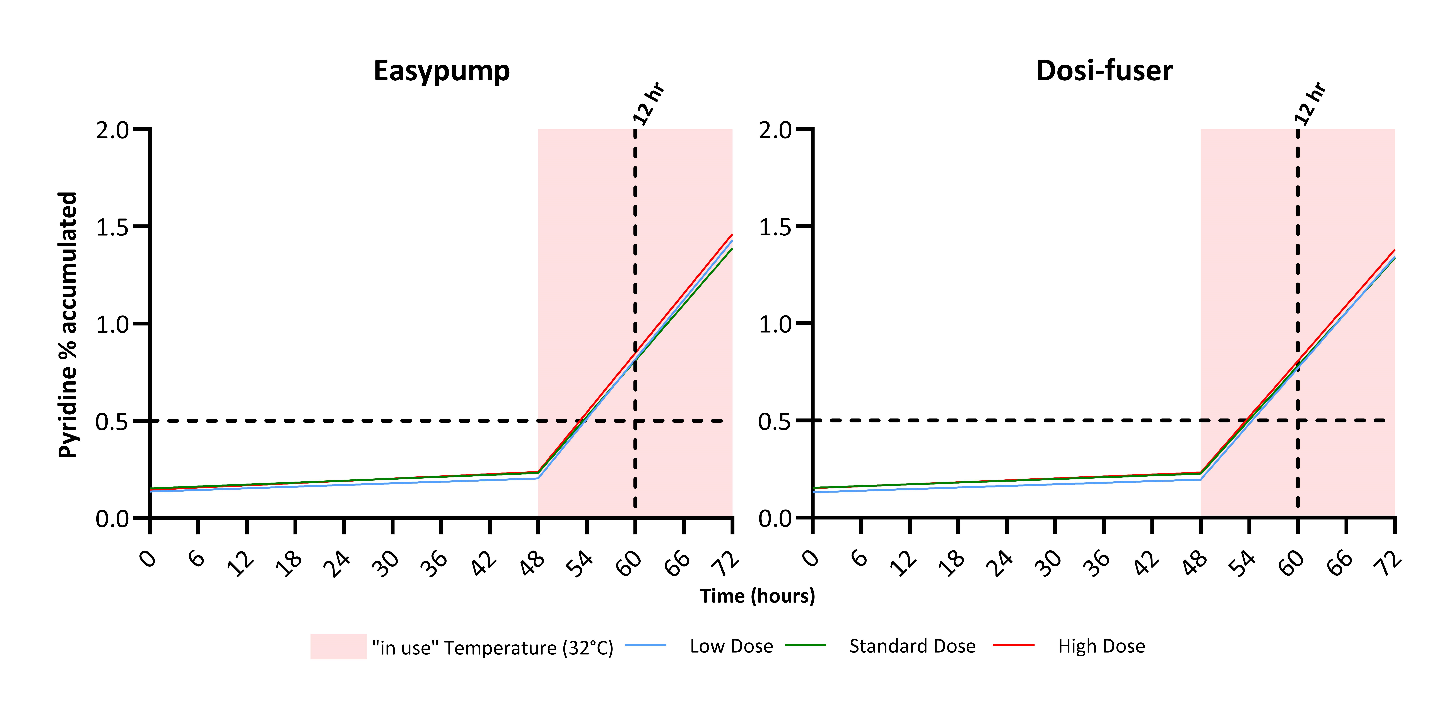


**Figure S6**. Regression model-simulated percentage of pyridine relative to baseline ceftazidime amount (%w/w) during 48 hours of fridge storage followed by 24 hours exposure to in-use temperature (32°C).


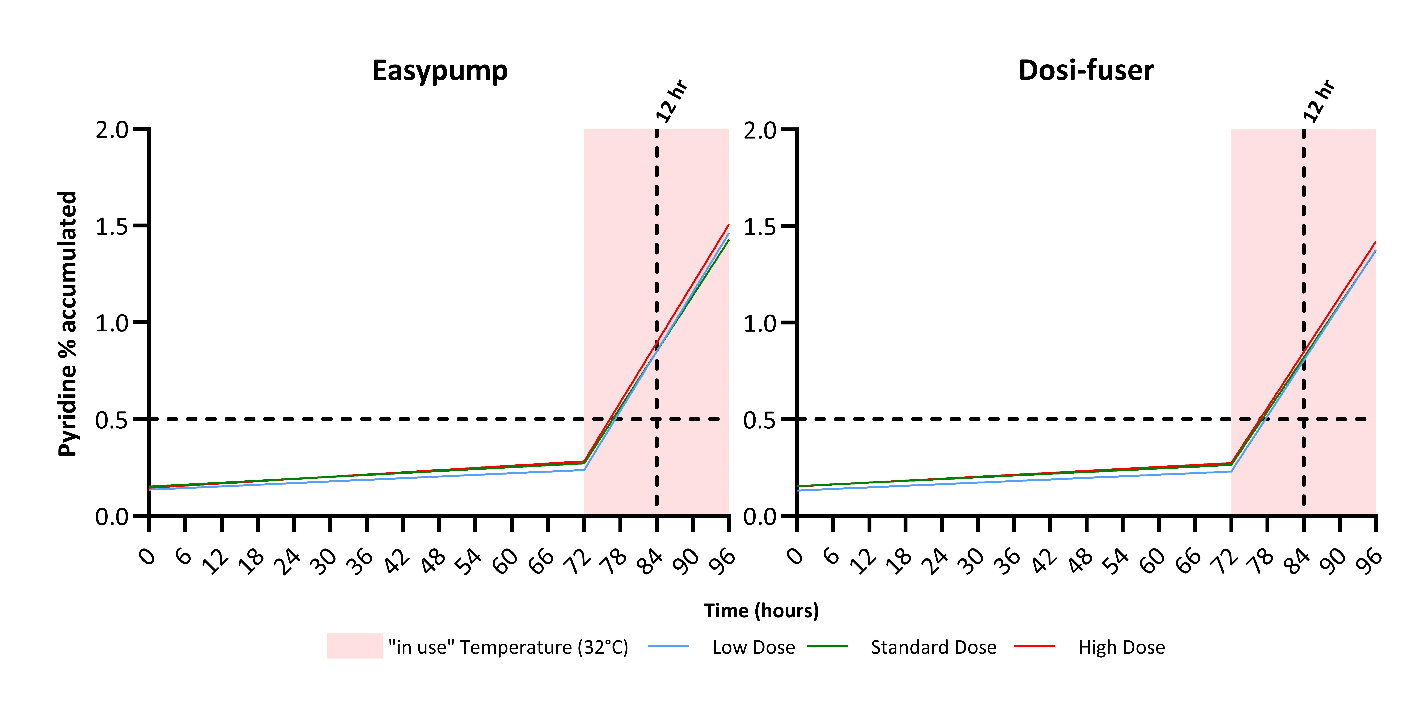


**Figure S7**. Regression model-simulated percentage of pyridine relative to baseline ceftazidime amount *(%w/w)* during 72 hours of fridge storage followed by 24 hours exposure to in-use temperature (32°C).
